# Salt‐Induced Stomatal Closure Suppresses Parasitism by *Phtheirospermum japonicum*

**DOI:** 10.1111/ppl.70657

**Published:** 2025-11-27

**Authors:** Clarissa F. Frederica, Masashi Asahina, Emi Yumoto, Louis J. Irving

**Affiliations:** ^1^ Graduate School of Science and Technology University of Tsukuba Tsukuba Japan; ^2^ Institute for Collaborative Biotechnologies University of California Santa Barbara Santa Barbara California USA; ^3^ University of Tsukuba Ibaraki Japan; ^4^ Advanced Instrumental Analysis Center Teikyo University Tochigi Japan; ^5^ Department of Biosciences Teikyo University Tochigi Japan; ^6^ Faculty of Life and Environmental Sciences University of Tsukuba Tsukuba Japan

**Keywords:** abiotic stress, facultative root hemiparasite, plant parasitism, salinity stress, stomatal conductance

## Abstract

In nature, parasitic plants may grow in marginal and stressful environments. However, the effects of concurrent abiotic stress, such as salinity, on parasitism remain underexplored, particularly in facultative root hemiparasites. Here, we examined the effect of salt on the relationship between the parasite *Phtheirospermum japonicum* and its host, *Arabidopsis thaliana* . Split‐root systems allowing separate application of salt treatment to hosts and parasites were used, enabling disambiguation of effects and the quantification of host‐to‐parasite salt transfer and nutrient abstraction rates. Host attachment increased parasite susceptibility to salt stress, due to a combination of enhanced salt uptake by the parasite and significant salt transfer from salt‐treated hosts. Salt treatment suppressed parasitic growth benefits, seemingly through a decrease in nutrient transfer rates. Follow‐up studies suggested that nutrient abstraction from the host was strongly tied to parasite stomatal conductance, which was attenuated by salt‐induced stomatal closure. Summarily, we propose that parasitism‐induced stomatal dysregulation led to salt accumulation within parasite leaves, which exacerbates salt effects, compromising its ability to abstract nutrients from its host. Our findings highlight the importance of local growing conditions and the presence of concurrent abiotic stressors in mediating the outcomes of the host–hemiparasite relationship.

## 1 Introduction

Parasitic plants (ca. 4750 spp.) abstract nutrients from their hosts, leading to improved parasite growth at the expense of the host (Nickrent 2020). They are a major threat to food security due to their impact on agriculture and can cause devastating losses to crop yields and economic profit (Rodenburg et al. 2016). In nature, parasitic plants and their hosts may occur in marginal environments that would subject them to one or more abiotic stresses (FAO 2007). However, while abiotic stress and plant parasitism have been studied extensively in isolation, few studies have explored how concurrent abiotic stress may affect the host–parasite relationship (Zagorchev et al. 2021).

Soil is considered ‘saline’ where the electrical conductivity (EC) of its saturation extract is greater than 4 dS m^−1^ at 25°C (approximate soil solution concentration of 40 mM NaCl), and it is estimated that approximately 10.7% of total global land is affected by salinisation (FAO 2024), with its prevalence expected to increase as a result of irrigation practices and climate change (Okur and Örçen 2020). Excess salinity can cause significant reductions in plant growth via the induction of multiple secondary stresses, including osmotic and oxidative stress, and ion toxicity when salt accumulates within plant leaf tissues (Munns and Tester 2008). Salt tolerance varies significantly between and within plant groups (James et al. 2008), with halophytic species possessing adaptations that allow them to grow under saline conditions without negative impacts on their growth. Salt tolerance mechanisms generally fall under two categories: plants may possess mechanisms to exclude salt or alternatively develop methods to safely sequester salt at higher concentrations. These mechanisms may include increased xylem salt export (Hauser and Horie 2010), suberisation or lignification of plant roots (Jbir et al. 2001; Neves et al. 2010; Oliveira et al. 2020), or the accumulation of compatible solutes and osmolytes (Horie et al. 2012) to overcome the reduced water potential of saline soils.

Salt stress can affect parasitism both pre‐ and post‐attachment to a host, although there are notably fewer studies characterising post‐attachment effects. At the pre‐attachment stage, salt may directly interfere with parasite seed germination or the production of haustoria initiation factors (Al‐Khateeb et al. 2003, 2005; but see Cochavi et al. 2018). Conversely, salt‐treated *Arabidopsis thaliana* hosts exhibited increased susceptibility to infection by parasite *Phelipanche ramosa* (Demirbas et al. 2013), although this has not been consistently observed in other studies. Post‐attachment, it is unclear if parasites may be affected by reductions in the quality of salt‐stressed hosts. As parasites connect to the host vascular system, parasitic plants may be exposed to high salinity even if they lack direct contact with saline soils as a result of host‐to‐parasite solute transfer (Frost et al. 2003; Veste et al. 2015; Zagorchev et al. 2018). However, the availability of host‐derived stress metabolites may be advantageous to the parasite (Veste et al. 2015). Frost et al. (2003) reported a dose‐dependent effect of salt, where the stem parasite *Cuscuta salina* exhibited reduced fecundity relative to controls when attached to *Beta vulgaris* hosts growing under low and moderate salinity, but had normal levels of fecundity under high salinity, suggesting that co‐occurring salt stress may suppress the parasite, but only under specific conditions. There is a scarcity of studies examining the impact of concurrent salinity stress on parasitism, with the existing literature limited to a narrow range of parasitic plant species, which comprise primarily, if not entirely, of obligate parasites. The effect of salinity on facultative root hemiparasites remains untested.

Unlike holoparasites, facultative root hemiparasites retain at least some photosynthetic ability, and are thought to be primarily parasitic for mineral nutrients and water, which they derive from the host xylem. Hemiparasitic haustoria are generally thought to lack ion selectivity (Seel and Jeschke 1999), which may render the parasite vulnerable to salt transfer from the host, but it is unknown how salt accumulation may affect the nutrient abstraction ability of hemiparasites, which are thought to rely on transpiration to abstract nutrients from their hosts (Marshall and Ehleringer 1990; Jiang et al. 2003). Sequestration of host‐derived salt has been shown to be important in maintaining the host–parasite water potential gradient in mistletoes (Veste et al. 2015) and some root parasites (Clermont et al. 2019). However, salinity has been reported to suppress plant stomatal conductance (Brugnoli and Lauteri 1991; Sharipova et al. 2022) through the accumulation of abscisic acid (ABA) in shoot tissue (Song et al. 2023). It is unknown if hemiparasites will respond to salt in a similar manner, with some species exhibiting insensitivity to exogenous ABA application (Lechowski 1996; Jiang et al. 2003), likely due to the high level of endogenous ABA in their leaf tissues relative to their hosts. While ABA insensitivity is thought to be an evolutionary adaptation for parasitism, it may be maladaptive in the presence of drought (Světlíková et al. 2018) or salinity stress, where stomatal behaviour may be crucial in stress tolerance. Jasmonates, which refer to a family of plant phytohormones known to be involved in long‐distance and interplant communication during pathogen attacks and herbivory, and more recently been shown to be involved in parasitic plant invasion response, are also involved in multiple abiotic stress signalling pathways, including salinity stress (Tamogami et al. 2012; Lee and Seo 2014; Wang et al. 2020). Further investigative studies with a greater range of parasitic species are needed to gain a broader understanding of how parasitism and salinity stress response interact.

In this study, we sought to address key research gaps relating to the long‐term effect of salt stress on host–hemiparasite relations. We grew the facultative root hemiparasite *Phtheirospermum japonicum* with an *Arabidopsis thaliana* host in a split root system, which allowed us to control salt supply to each plant individually, and to quantify host‐to‐parasite resource and salt transfer. We hypothesised that salt supplied to the host would accumulate in the parasite, resulting in suppressed parasite growth and a decline in overall parasitism in the system. As hypothesised, we observed reduced parasite growth when salt was supplied to either *Phtheirospermum* or Arabidopsis, with commensurate reductions in resource transfer rates. Follow‐up experiments revealed that parasite stomatal conductance was suppressed by salt, and exogenous ABA application demonstrated that parasite stomatal closure could suppress host‐to‐parasite resource transfer.

## 2 Materials and Methods

**FIGURE 1:**
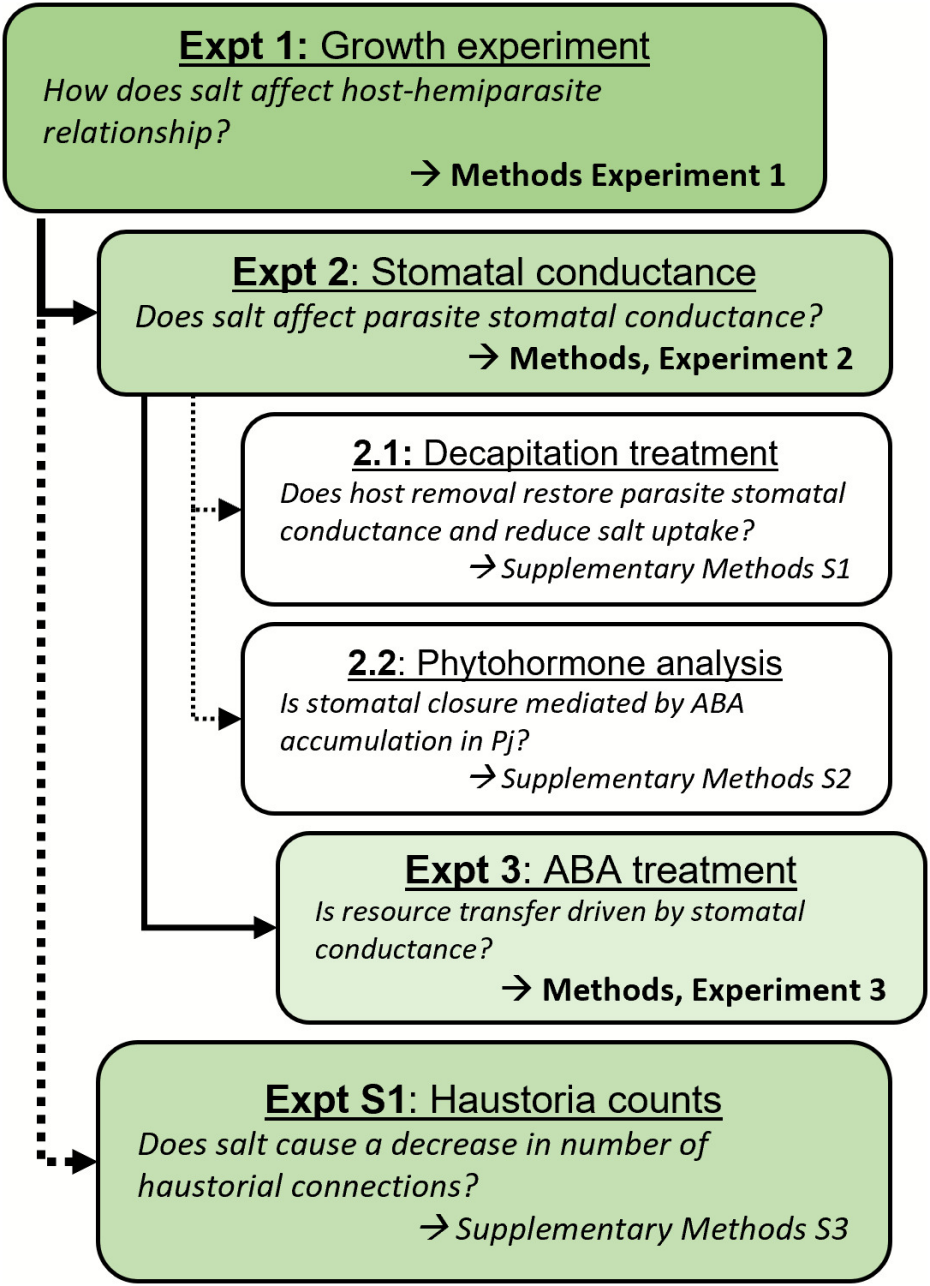
Experimental road‐map.

In this paper, we report the findings of a series of experiments studying the effect of salinity on the relationship between host *Arabidopsis thaliana* and its root hemiparasite *Phtheirospermum japonicum*. An experiment roadmap is outlined in Figure 1, which includes the sequence of experiments reported here, as well as the primary objective or research question of each study. The detailed methods of any side investigations can be found in the Supplementary Methods as indicated in Figure 1.

### 2.1 Plant Material

*Phtheirospermum japonicum* (Family: Orobanchaceae) is a facultative root hemiparasite native to East Asia. Despite being a model parasitic plant for haustorial developmental studies, ecological data on *Phtheirospermum* are scarce, with little information available regarding its commonly associated host(s) and its naturally occurring biomes. Limited descriptions indicate that *Phtheirospermum* is generally found in grassland and forest fringes, as well as near reservoir basins. *Phtheirospermum* is considered to be a generalist, with the ability to parasitise a wide range of host species, including Arabidopsis (used in this study) as well as grass and legume species.

### 2.2 Experiment 1: Growth Experiment

**FIGURE 2:**
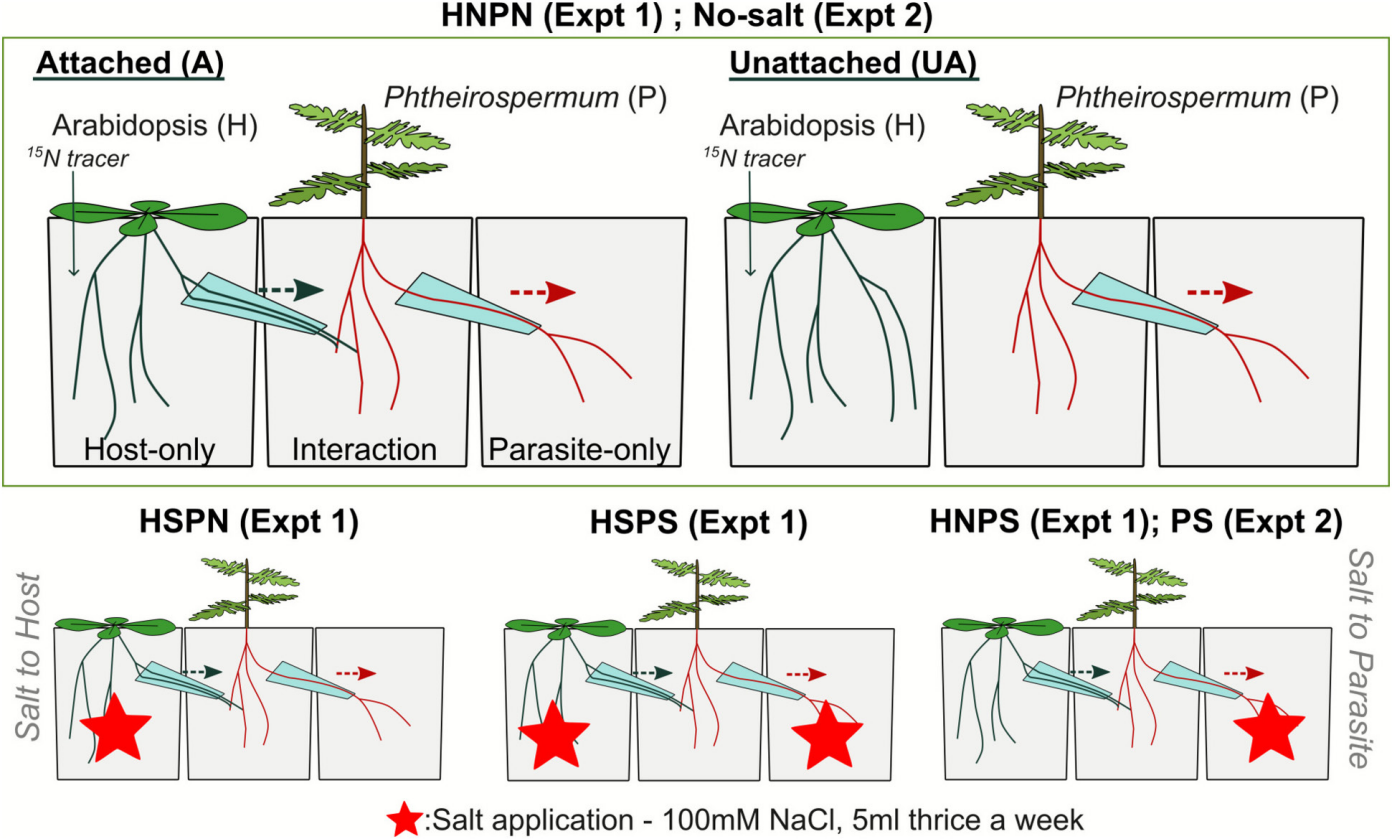
(a) Split‐root system setup and (b) salt treatments used in Experiment 1 and 2. This figure does not show the full array of treatment groups—only attached salt treatments are reflected.

This experiment aimed to characterise the effect of salt supply to the host and parasite on the parasitic relationship. A split‐root system allowing independent salt supply to the host and parasite was created using three square pots (A‐PET material, dimensions approx. 6 × 6 × 6 cm) as shown in Figure 2. A total of 64 replicates were set up. A 3‐cm‐long piece of 12 mm‐diameter straw connected the parasite‐only and interaction compartments. In half of the replicates, a root bridge was constructed between the host‐only and interaction compartments using a 5 mL pipette tip trimmed to approximately 4 cm long. This root bridge facilitated host root growth into the interaction compartment, allowing haustoria formation in the ‘Attached’ groups. The smaller tip opening was located in the interaction compartment to deter parasite root growth into the host‐only compartment. Pots were filled with vermiculite and placed in trays. After the experiment, root bridges were inspected, with no evidence of unwanted root crossover found.

*Arabidopsis thaliana* seeds were cold‐stratified on moist filter paper at 4°C for 2 days prior to planting. *Phtheirospermum* seeds were sown directly onto the vermiculite in the interaction compartment. Cold‐stratified Arabidopsis seeds were suspended in dH_2_O and pipetted directly onto the vermiculite in the host compartment. Trays were covered with plastic wrap for 7 days to promote seed germination. Plants were grown under LED tube lights (150 μmol m^−2^ s^−1^ at plant height) under short‐day conditions (8/16 h light/dark conditions, 25°C/22°C day/night) to extend the vegetative period of Arabidopsis hosts. Plants were trimmed to a single host or parasite per replicate.

Arabidopsis and *Phtheirospermum* seedlings were fed thrice a week with Hoagland's solution (Hoagland and Arnon 1950), commencing from 7 and 10 days after planting, respectively. The volume of nutrient solution given to Arabidopsis per feeding was increased in two increments: 2 mL was given from 7 days after planting (dap), 5 mL from 14 dap, then finally 10 mL per feeding from 28 dap until harvest. *Phtheirospermum* feeding volume was increased once: 2 mL from 10 dap, then 5 mL per feeding after 14 dap until harvest. Salt treatments began 35 dap and consisted of thrice‐a‐week applications of 5 mL of 100 mM NaCl solution to the respective host and/or parasite‐only compartments as indicated on Figure 2. The experiment followed a 2 × 2 × 2 factorial design with attachment (unattached, ‘UA’ vs. attached, ‘A’), host (H) salt status and parasite (P) salt status (no salt, ‘N’ vs. salt, ‘S’) as fixed factors. Salt treatment was provided at the same time as nutrients for 5 weeks before destructive harvesting (70 dap). Deionised water was provided to the bottom of the tray as needed to prevent desiccation throughout the growing period.

Two days before harvest, 1 mg of ^15^N isotope tracer was provided to each Arabidopsis host plant in the form of double‐labelled ammonium nitrate (^15^NH_4_^15^NO_3_) to quantify host‐to‐parasite nutrient transfer. The next day (1 day before harvest), linear electron flow (LEF) of *Phtheirospermum* was measured on the fifth leaf counted basipetally using the RIDES 2.0 protocol on a MultiSpeQ (PhotosynQ Inc). On the day of harvest, the same *Phtheirospermum* leaf was cut and placed into 10 mL of methanol (containing 10 mg L^−1^ MgCO_3_) for chlorophyll quantification. Three small sections of rosette leaves were randomly collected from each Arabidopsis plant for chlorophyll quantification. Chlorophyll was quantified spectrophotometrically using the equations of Porra et al. (1989).

Leaves directly adjacent to those harvested for chlorophyll quantification were used for fresh/dry weight correction. Arabidopsis and *Phtheirospermum* were excised at the shoot base, wrapped in foil, and dried at 70°C for at least 1 week before their dry weights were measured. Parasite samples were milled into powder for subsequent analyses. Host and parasite shoot tissues were digested using a micro‐Kjeldahl method and their sodium concentration measured by inductively coupled plasma mass spectrometry (ICPS‐8100, Shimadzu, Tokyo, Japan), while shoot nitrogen (N) concentration was quantified spectrophotometrically using Nessler reagent (Mcdonald 1978). Parasite ^15^N abundance was measured by isotope ratio mass spectrometry (EA Isolink CN IRMS System, ThermoFisher Scientific). The ^15^N natural abundance of unlabelled *Phtheirospermum* parasites was determined to be 0.3677 atom% (data not shown). Host‐to‐parasite ^15^N transfer was estimated from the ^15^N atom% excess. *Phtheirospermum* from unattached groups had negligible ^15^N excess, confirming that leakage of nutrients was not an issue.

### 2.3 Experiment 2: Stomatal Conductance Time‐Series

In Experiment 1, we found that (1) an increase in salt uptake was observed in salt‐supplied parasites that were attached to a host, and (2) salt supply to the parasite and the host resulted in a decrease in host‐to‐parasite nutrient (^15^N) transfer. As transpiration is generally thought to provide the motive force for host to parasite resource transfer in hemiparasites, we hypothesised that increased stomatal conductance (*g*_*s*_) may explain observed increases in salt uptake in the attached, salt‐supplied parasites. However, as salinity has been shown to cause declines in plant stomatal conductance rates (Brugnoli and Lauteri 1991; Sharipova et al. 2022), we also hypothesised that the accumulation of shoot sodium may cause a reduction in parasite stomatal conductance in the later stages of salt treatment. To test this, we measured daytime *g*_*s*_ in host‐attached (‘A’) or unattached (‘UA’) *Phtheirospermum* parasites over a 42‐day salt treatment period, under no salt conditions (‘No‐salt’) or where salt was supplied to parasite roots (PS).

Plants were grown as described in ‘Experiment 1’ with smaller boxes (dimensions: 5.5 × 5.5 × 4 cm). Arabidopsis plants were supplied 5 mL of nutrient solution three times per week from 14 dap to the end of the experiment to adjust for the smaller root box size. Nutrient volumes and frequency were kept the same for *Phtheirospermum* as described in Experiment 1. From 35 dap, 5 mL of 100 mM NaCl was supplied to *Phtheirospermum* plants in the PS groups thrice a week for 6 weeks.

*Phtheirospermum* leaf stomatal conductance (*g*_*s*_) was measured 10, 20, 30 and 40 days after the start of salt treatment using a SC‐1 leaf porometer (Decagon Devices), with measurements taken from the abaxial surface of the fourth leaf counted basipetally approximately 4 h after the start of the light period (12:00 PM). Trays were watered generously at least 2 h before *g*_*s*_ was measured. Forty‐two days after the start of salt treatment (77 dap), Arabidopsis and *Phtheirospermum* were destructively harvested. To validate the findings from Experiment 1, *Phtheirospermum* dry weight and chlorophyll concentration were measured (as described above). Shoot sodium and potassium concentrations were also quantified. During destructive harvesting, the fresh weights of three to four randomly selected leaves from *Phtheirospermum* were measured for the calculation of relative water content (RWC). RWC was determined by dividing the difference between leaf fresh and dry weights by the fresh weights.

Two ancillary studies were carried out alongside Experiment 2 to test if (1) increased parasite salt accumulation was related to the elevated parasite transpiration rates associated with host attachment, and (2) to examine if parasite stomatal closure observed under salt conditions was accompanied by changes in leaf phytohormone concentrations. While abscisic acid (ABA) was the primary focus of the study due to its well‐reported function in leaf stomatal behaviour, jasmonic acid (JA) and its derivatives and cytokinins were also simultaneously measured to obtain a comprehensive view of the phytohormone response within *Phtheirospermum*. The detailed methodology of these two studies can be found in Methods S1 and S2, respectively.

### 2.4 Experiment 3: Effect of Exogenous ABA‐Treatment on ^15^N Transfer

In Experiments 1 and 2, we found reductions in host‐to‐parasite ^15^N transfer and parasite stomatal conductance, respectively, in salt‐treated groups, and hypothesised that stomatal closure can limit resource flux in our studied parasite. To test for a causal relationship between parasite transpiration rate and host‐to‐parasite resource transfer, we applied differing concentrations of abscisic acid (ABA) to the leaves of *Phtheirospermum* and quantified ^15^N transfer from the Arabidopsis hosts.

Plants were set up as described in Experiment 2, with a total of 40 replicates, all allowing parasitic attachment. Nutrient feeding volumes and frequency to Arabidopsis and *Phtheirospermum* were identical to Experiment 2, although salt treatments were not given. Plants were grown for a total of 9 weeks. One day before harvest (62 dap), each replicate was randomly assigned to one of four groups. At 8:00 AM, *Phtheirospermum* leaves were painted on both abaxial and adaxial surfaces with ABA solutions at 10^−4^, 10^−5^, and 10^−6^ M. For the control group, leaves were painted with dH_2_O. Daytime *g*_*s*_ was measured thrice at 2.5, 3.5, and 5 h after ABA application. Different leaves were selected at random for *g*_s_ measurements at each time point. Six hours after ABA treatment, 1.0 mg of ^15^N (as ^15^NH_4_^15^NO_3_) was fed to each Arabidopsis host. Plants were harvested 24 h after ^15^N feeding, separated into leaves and stem tissue, and prepared for ^15^N quantification as described in Experiment 2.

### 2.5 Statistical Analyses

Statistical analyses were performed on R version 4.5.0 (R Core Team 2023). ANOVA tables are reported in the Supporting Information.

#### 2.5.1 Experiment 1

Host and parasite shoot biomass, shoot N concentration, chlorophyll concentration, and parasite leaf electron flow (LEF) were analysed by three‐way ANOVA with attachment, parasite salt status, and host salt status as fixed factors. Where significant interactions were found, pairwise comparisons of estimated marginal means (using the emmeans R package; Lenth et al. 2025) or simple main effects were used for further analyses. If necessary, data were transformed prior to analysis to achieve normally distributed residuals and homoscedasticity. The ^15^N content of attached *Phtheirospermum* parasites was analysed using Welch's one‐way ANOVA with treatment group as a fixed factor, followed by Games‐Howell post hoc analysis to identify differences between groups (rstatix package; Kassambara 2023). Arabidopsis and *Phtheirospermum* shoot sodium concentration were separately analysed using Mann–Whitney *U* non‐parametric tests to test for differences between unattached and attached groups at each salt treatment (HNPN, HSPN, HNPS, or HSPS).

#### 2.5.2 Experiment 2

Parasite *g*_*s*_ was analysed using repeated measures analysis of variance (RM‐ANOVA; using the aov function) with day as a within‐subjects factor, and attachment and parasite salt status as between‐subjects factors. Huynh‐Feldt sphericity corrections were used where assumptions of sphericity were violated.

#### 2.5.3 Experiment 3

A three‐parameter asymptotic regression model was fitted using the drm command in the statforbiology R package (Onofri 2024) to parasite ^15^N content plotted against parasite stomatal conductance (averaged over the three time‐point readings).

## 3 Results

### 3.1 Experiment 1: Salt Supply to Arabidopsis or *Phtheirospermum* Suppressed Parasitism

**FIGURE 3:**
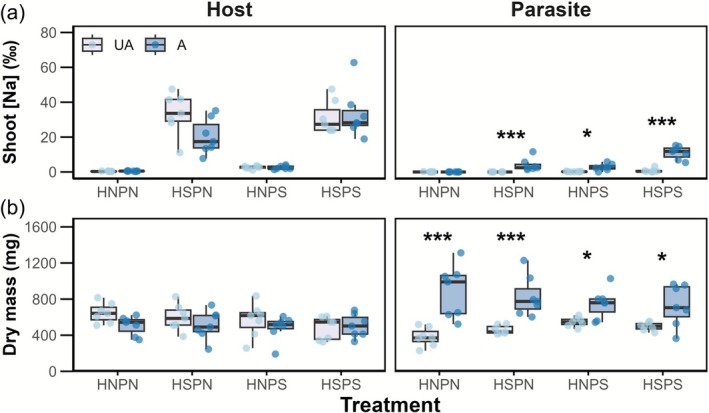
Host‐to‐parasite salt transfer occurs when *Phtheirospermum* is attached to salt‐treated hosts. Increases in parasite dry mass associated with parasitic benefit are less prominent when the parasite is salt‐treated (Experiment 1). *Arabidopsis* and *Phtheirospermum* (a) shoot sodium concentration and (b) dry mass in unattached (UA) and attached (A) boxes under each treatment group. H/P = Host/Parasite, N/S = No‐salt/Salt. Asterisks indicate significant differences between UA and A groups (*/***p < 0.05, 0.001, respectively, pairwise comparisons). *n* = 7.

In the absence of parasitism (unattached, ‘UA’ groups), salt‐supplied Arabidopsis plants expectedly contained high Na^+^ concentrations (32.3‰ ± 2.79‰), while salt‐supplied *Phtheirospermum* plants had a mean [Na^+^] of 0.45‰ ± 0.21‰ (Figure 3a). This difference was presumably due to the greater exposure of Arabidopsis plants, which received salt solution directly in their rooting chamber, as opposed to *Phtheirospermum*, which received salt in the parasite‐only chamber (Figure 2).

Host attachment led to a three‐fold increase in shoot [Na^+^] in salt‐supplied *Phtheirospermum* connected to no‐salt Arabidopsis hosts (HNPS), suggesting increased salt uptake by *Phtheirospermum* roots under parasitic conditions (*p* = 0.025, UA vs. A, Mann–Whitney *U* test), while no‐salt parasites connected to salt‐supplied hosts (HSPN) had a mean [Na^+^] of 4.02‰ ± 1.37‰ compared to their background trace levels of Na^+^ in unattached parasites, indicating host‐to‐parasite salt transfer (*p* = 0.001). Attached HSPS parasites exhibited a 10‐fold increase in shoot [Na^+^] relative to UA plants (*p* < 0.001), which was likely the combined result of enhanced salt uptake and host salt transfer.

Salt did not have an impact on unattached Arabidopsis or *Phtheirospermum* biomass (Figure 3b, Table S1). Arabidopsis biomass was only marginally reduced by parasite attachment (*p* = 0.062), while host attachment had a positive effect on *Phtheirospermum* biomass: host‐attached *Phtheirospermum* was always found to be heavier than unattached *Phtheirospermum*, irrespective of salt treatments (Figure 2b). However, the increase attributed to host attachment was smaller where *Phtheirospermum* was supplied salt (UA vs. A PS, +42%, *F* = 12.053***), compared to the no‐salt parasites (UA vs. A PN, +105%, *F* = 58.305***, simple main‐effects).

**FIGURE 4:**
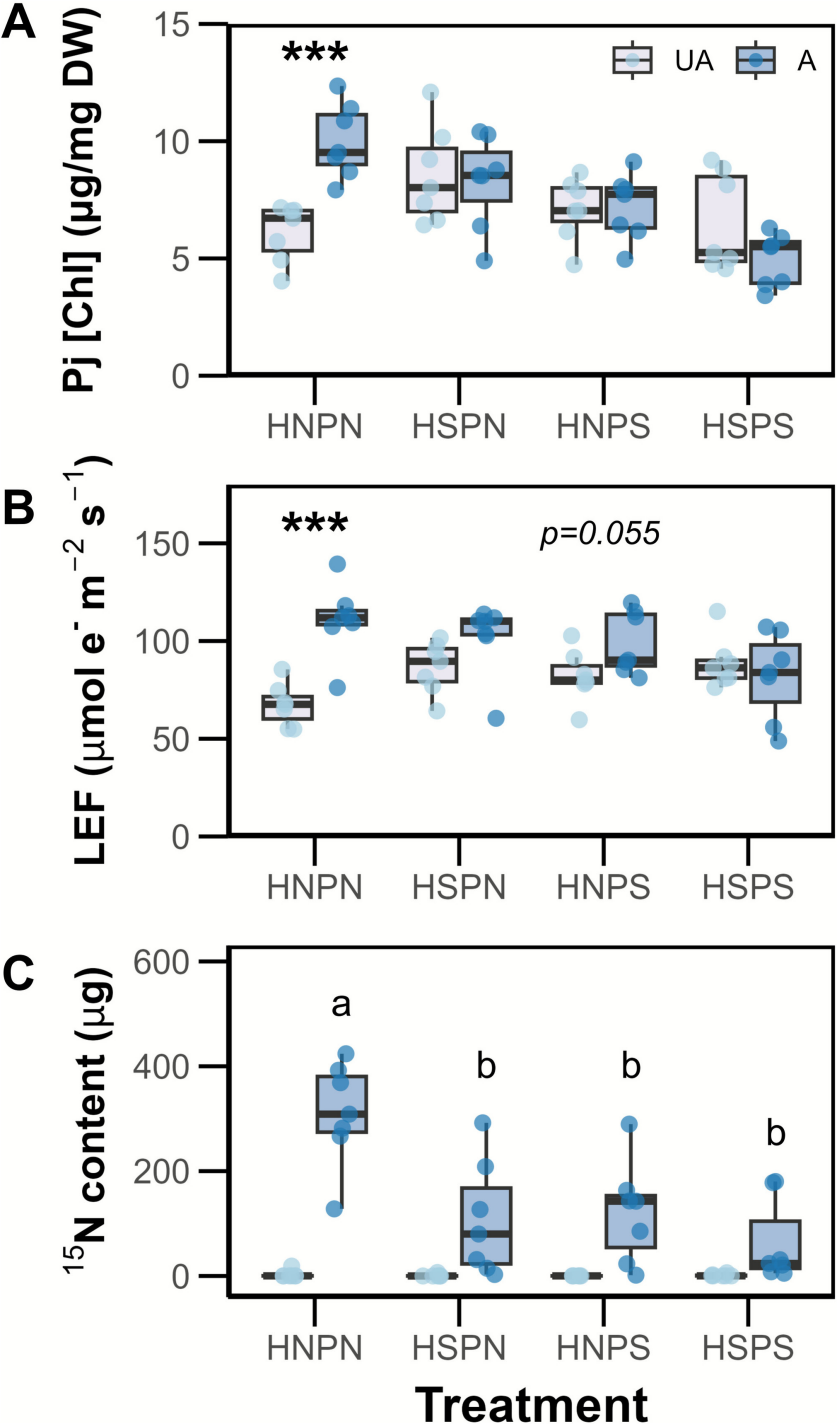
Salt treatment to either host Arabidopsis and/or parasite *Phtheirospermum* leads to absent or less prominent parasitic benefits, as well as reduced host‐to‐parasite resource flux (Experiment 1). *Phtheirospermum* (a) chlorophyll concentration, (b) linear electron flow (LEF) and (c) ^15^N content in unattached (UA, light blue) and attached (A, dark blue) boxes under each treatment group. H/P = Host/Parasite, N/S = No‐salt/Salt. Asterisks indicate significant differences between UA and A groups (in a, b; ***p < 0.001, pairwise comparisons), lowercase letters indicate significant differences between attached treatment groups (in c, Games‐Howell's post hoc test). *n* = 7.

This trend was maintained across the other parameters measured: host attachment under HNPN conditions led to a + 64% increase in *Phtheirospermum* chlorophyll concentration (*F* = 16.156***; Figure 4a, Table S2) and a corresponding increase in parasite linear electron flow (*F* = 25.239***; Figure 4b), indicating a strong positive effect of parasitism under no‐salt (HNPN) conditions. However, these increases were either absent or less pronounced in the salt treatment groups: No differences were found in chlorophyll concentrations in any of the salt treatment groups (*p* > 0.05), and only a marginal increase in LEF was observed where either Arabidopsis or *Phtheirospermum* were salt‐treated (HSPN, *p* = 0.083; HNPS, *p* = 0.055, pairwise comparisons). No increases in parasite LEF were observed in the HSPS group (*p* > 0.05). Attachment had no effect on parasite shoot nitrogen concentration (see Figure S1, Table S3).

Attached *Phtheirospermum* ^15^N content differed between treatments (*F* = 8.456, *p* = 0.002, Welch's one‐way ANOVA; Figure 4c), with ^15^N transfer being highest in the no‐salt treatment (HNPN: 310 μg). When either the host or parasite was salt‐treated (HSPN, HNPS), the parasite ^15^N content was reduced by similar amounts (−67%) relative to the no‐salt group, while a −81% reduction was observed where both host and parasite were salt‐treated (HSPS). The differences between the three salt treatments were not statistically significant.

### 3.2 Experiment 2: Salt Reduced Parasite Stomatal Conductance Rates

**FIGURE 5:**
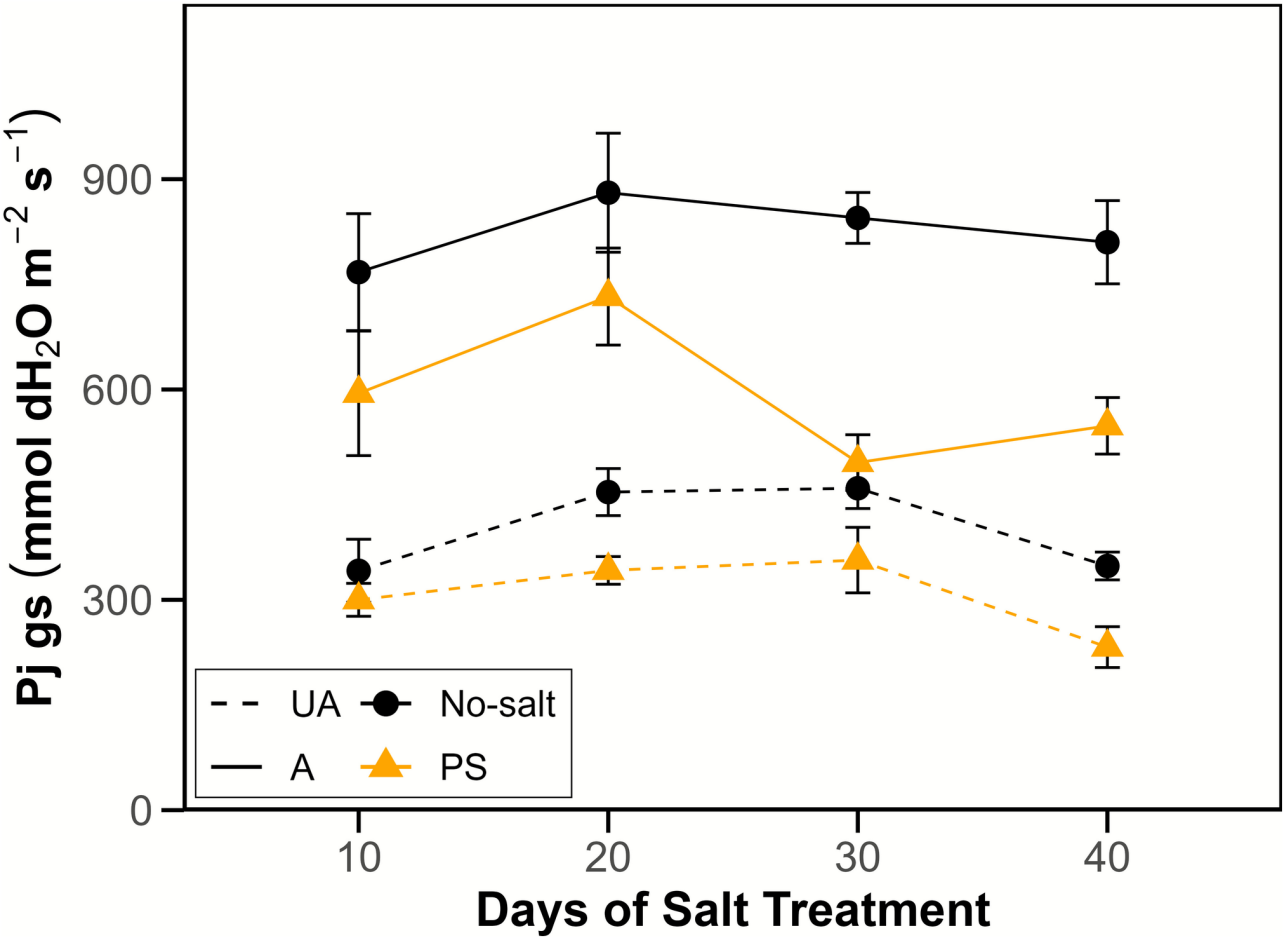
Salt treatment resulted in declines in *Phtheirospermum* daytime stomatal conductance (Experiment 2). *Phtheirospermum* daytime stomatal conductance (*g*_*s*_) on days 10, 20, 30 and 40 of a 42‐day salt treatment period. Dashed and solid lines indicate unattached (UA) and unattached (A) parasites, colour indicates salt treatments (black = no‐salt, orange = parasite‐salt, PS). Points and error bars indicate mean ± standard error. *n* = 8.

Attached *Phtheirospermum* parasites maintained a higher *g*_s_ than unattached plants on all days (*F* = 35.88***; Figure 5), while salt‐supplied *Phtheirospermum* (PS) possessed lower *g*_s_ (−25%) than no‐salt plants regardless of attachment status (*F* = 170.46***). An interaction between attachment and treatment was reported (*p* = 0.011, Table S4), but this may be attributable to the magnitude of absolute differences in UA versus A *g*_s_ within no‐salt and PS groups.

As seen in Experiment 1, increases in parasite dry weight and chlorophyll concentrations associated with host attachment were reduced and absent, respectively, in the PS treatments (see Table S5 for means and statistical analyses). Leaf relative water content (RWC) did not differ between UA no‐salt and UA PS plants (*p* = 0.450, simple main effects) but was increased in A PS parasites relative to UA PS plants (*F* = 21.686***). Attachment led to increases in parasite leaf sodium and potassium levels in PS treatments (*p* < 0.001, *p* = 0.002, respectively; Figure S2) but had no effect under no‐salt conditions.

### 3.3 Experiment 3: Exogenous ABA Application Reduced ^15^N Transfer

**FIGURE 6:**
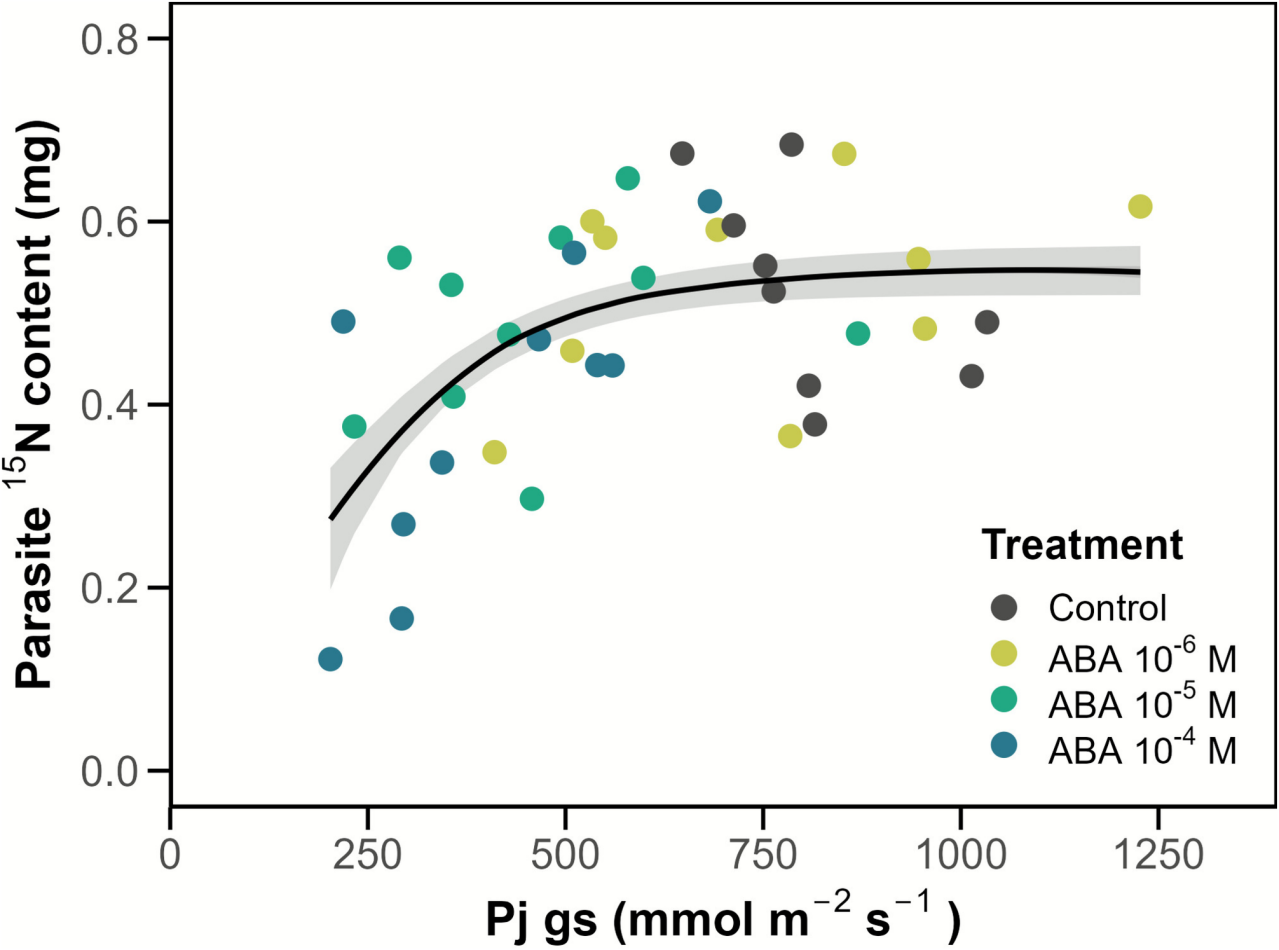
^15^N resource flux is reduced following exogenous ABA application and parasite stomatal closure (Experiment 3). Dose–response curve of *Phtheirospermum* ^15^N content plotted against parasite stomatal conductance, *g*_s_ (solid black line), with standard error indicated in grey. Points represent individual replicates for each treatment (indicated by different colours). Results of curve is reported in Table 1.

Parasite *g*_s_ declined within 5 h of ABA application at concentrations 10^−5^ and 10^−4^ M, but not at 10^−6^ M (Figure S3) (Jiang et al. 2003). Stomatal conductance did not differ between control plants and those supplied with the lowest ABA levels (10^−6^ M, *p* = 0.991), but was 45% lower in the 10^−5^ and 10^−4^ M treatments (*p* < 0.001). Parasite ^15^N content increased with stomatal conductance towards a maximum value (Figure 6, Table 1). The non‐zero exponent of the *g*_s_‐parasite ^15^N content curve suggests that host‐to‐parasite ^15^N transfer was suppressed by ABA‐induced stomatal closure.

**TABLE 1:** Results of *g*_s_‐parasite ^15^N content curve model depicted in Figure 5.

	Estimate	SE	*t* value	*p* value
*Y*‐intercept	−0.345	0.455	−0.759	0.453
Exponent (×10^−3^)	5.67	1.80	3.154	0.003
Plateau	0.547	0.028	19.781	< 0.001

## 4 Discussion

In this paper, we demonstrated that salt treatment reduced parasitic benefit, seemingly through a decline in host‐to‐parasite resource transfer as a result of salt‐induced stomatal closure. Host attachment promoted the salt susceptibility of the parasite through host‐to‐parasite salt transfer and elevated transpiration rates, which in turn led to the increased accumulation of salt within parasite leaf tissues.

### 4.1 Salt Treatment Suppressed Parasitism and Resource Transfer

In the absence of salt, attachment to an Arabidopsis host led to a doubling in attached parasite biomass, and similar increases in chlorophyll concentration and LEF. Increases in the mass of attached *Phtheirospermum* parasites were likely due to a combination of increased parasite photosynthetic rates and resource transfer from the host (Seel et al. 1993; Hibberd and Jeschke 2001; Irving et al. 2019; Frederica and Irving 2024). Salt supply to either the host or the parasite led to a reduction in parasitic growth benefits, with supply to both plants causing the greatest suppression. Commensurate reductions in both LEF and ^15^N transfer may explain reductions in growth rate, presumably due to decreased host resources (Figure 4c) and carbon fixation rates due to lowered stomatal conductance (figure 5 in Gago et al. 2016). Salt had little effect on the growth of unattached Arabidopsis and *Phtheirospermum*, suggesting that the noted declines in parasitic benefit were not a direct effect of salt exposure on individual plant performance.

### 4.2 Host to Parasite Salt Transfer and Increased Susceptibility to Salt in Attached Parasites

Parasites attached to salt‐treated Arabidopsis plants exhibited increased leaf sodium content, confirming host‐to‐parasite salt transfer. Attached HSPN parasites contained substantially higher shoot sodium concentrations relative to unattached salt‐treated (PS) *Phtheirospermum*, suggesting that the parasite has a weak ability to defend or select against host‐derived salt. Attached *Phtheirospermum* parasites displayed increased salt susceptibility relative to their unattached counterparts. As sodium uptake and xylem loading are thought to be passive, with active efflux mechanisms (Maathuis et al. 2014), sodium accumulation in parasite shoots was presumably due to the higher transpiration rates resulting from attachment. This was further indicated through a host decapitation treatment conducted 10 days into salt treatments during Experiment 2 (described in Methods S1, see host‐free attached (HFA) groups in Figure S4), which showed subsequent restoration of parasite stomatal conductance to values comparable to unattached plants within 10 days, and a final shoot sodium concentration that was 45% lower than salt‐treated parasites with intact hosts (Figure S2).

Profligate transpiration has been observed in other hemiparasites (Marshall and Ehleringer 1990; Lechowski 1996) and is hypothesised to be the primary mechanism driving nutrient abstraction from their hosts. However, our findings suggest that it may also promote parasite root uptake and shoot translocation of both nutrients and, in the case of salt, potential toxicants. Smith and Stewart (1990) attributed the accumulation of potassium in *Striga* shoot tissue to their high transpiration rates, although further studies would be required to evaluate the relative contribution of potential increases in parasite (non‐host) nutrient uptake to improved parasite performance following host attachment. Increased salt uptake may act as a double‐edged sword in the context of hemiparasite relations. On one hand, salt may play an osmotic role in plants (Wege et al. 2017), and its accumulation may represent an energetically favourable method to maintain a water potential gradient with the host. Analogously, the accumulation of host‐derived sucrose and mannitol has been frequently observed in other hemiparasites (Veste et al. 2015; Clermont et al. 2019). However, our data suggest that the unregulated transfer of solutes from the host may expose the parasite to high concentrations of ions or other potentially toxic compounds.

### 4.3 Salt Treatment Reduced Parasite Stomatal Conductance, Which Impeded Parasite Resource Flux

Direct salt supply to *Phtheirospermum* caused a substantial decrease in parasite stomatal conductance rates (Figure 5), presumably due to the accumulation of sodium within leaf tissues (Figure 3a), resulting in stomatal closure (Sharipova et al. 2022). Consistent with this, attached PS plants exhibited increased leaf relative water content in Experiment 2, suggesting reduced evaporative water loss (Table S5). Stomatal closure is typically induced by the accumulation of ABA that is either endogenously produced within the leaves or translocated from plant roots (Bharath et al. 2021). However, some hemiparasites have been shown to exhibit abnormal stomatal behaviour, such as the maintenance of open stomata even under drought conditions (Světlíková et al. 2018), which was attributed to ABA insensitivity. High levels of endogenous ABA have been reported in attached parasitic plants (Lechowski 1996; Jiang et al. 2004). Our third experiment confirmed that *Phtheirospermum* is similar in its relative insensitivity to ABA; exogenous ABA application at a concentration of 10^−6^ M—a concentration which would trigger stomatal closure in most non‐parasitic species (Jiang et al. 2004)—failed to elicit a stomatal closure response, although higher ABA concentrations could induce stomatal closure (Figure S3), which subsequently resulted in reduced host‐to‐parasite ^15^N flux (Figure 6).

Phytohormone analysis revealed that while ABA concentrations were elevated in attached *P. japonicum* leaves, there was no effect of salt treatment (Figure S5). Increases in parasite ABA concentrations have been reported in multiple root hemiparasite species, including *Rhinanthus minor* (Jiang et al. 2004), *Melampyrum arvense* (Lechowski 1996), as well as our studied parasite, *Phtheirospermum* (Kokla et al. 2022). ABA concentrations in this study were significantly higher (approximately 4 [no host], 5 nmol g^−1^ [with host]) than those reported by Kokla et al. (2022) (approximately 0.3 [no host], 1 nmol g^−1^ [with host]), which we attribute to differences in plant material (mature leaves versus whole seedlings) and weight corrections (dry vs. wet mass). Increases in parasite leaf concentrations of jasmonic acid (JA) and its derivatives were noted in response to salt supply to either plant, but particularly where Arabidopsis was salt‐treated, surprisingly under both attached and unattached conditions. Speculatively, we hypothesise that salt‐stressed Arabidopsis host may have been producing volatile organic compounds (VOCs), which elicited JA‐signalling responses in their neighbouring *Phtheirospermum* plants regardless of their attachment status (Figure S5—see JA, JA‐Ile). JA is known to be associated with plant resistance to abiotic stress conditions, including activation of antioxidant systems (Smith et al. 2009; Wang et al. 2020), and has been implicated in stomatal closure under elevated CO_2_ (Geng et al. 2016) and drought stress responses (Savchenko et al. 2014), frequently in tandem with ABA. While it is plausible that JA signalling was involved in the stomatal closure of *Phtheirospermum*, its role is more likely to be indirect (Zamora et al. 2021).

Put together, we propose that the inhibition of parasite transpiration due to stomatal closure is the primary mechanism of salt‐induced parasitic suppression. Increased transpiration and/or host‐derived salt transfer in parasitic *Phtheirospermum* exacerbate the effects of salt on the parasite, resulting in eventual stomatal closure and a loss of motive force for the parasite to abstract nutrients from the host and decreased parasitic benefit in the long term. Note that there may be other mechanisms acting in tandem (discussed in the next section), although we postulate that salt‐induced stomatal closure may be the primary mechanism of suppression.

### 4.4 Other Considerations

While our experiments were designed to allow haustorial formation prior to the start of salt treatments, it is possible that new haustoria formation may have been impeded by salt treatments, resulting in fewer physical connections with its host, and thereby limiting nutrient transfer rates. We conducted a time‐series of haustoria counts under control or salt treatment conditions (Methods S1, Figure S6), which showed no apparent effect of salt on the rate of haustoria formation or maturation (Figure S7). This suggested that the ability of *Phtheirospermum* to attach to its host was unaffected by salt application. However, haustoria counts could only be obtained up to 15 days due to significant plant mortality on the slant‐boards, as opposed to our prior growth experiments where the salt treatments were carried out for 35–42‐days. It is possible that salt may have suppressed haustoria production over this longer period, as statistical analyses showed a marginal effect of salt treatment (Figure S7).

The impact of salt stress on the relationship between a hemiparasite and its host is likely influenced by numerous factors besides host compatibility, such as host and parasite salt tolerance. Salt‐tolerant parasite species are more likely to have a neutral response towards imposed salinity stress, although Frost et al. (2003) showed that *Cuscuta salina* derived less benefit from host attachment and had lower fecundity under intermediate (62.5, 125 mM) but not high (250 mM NaCl) salinity levels compared to controls. Their observed results are likely a product of host response rather than a parasite one: some form of partial host resistance may occur under low to moderate levels of salt, but under high levels of salt, host defences may be impaired or stress‐related metabolites may be exhausted (Zagorchev et al. 2021), resulting in improved parasite performance. This suggests that the parasite's response to salt may be less important than that of the host, as the resource acquisition strategy of the hemiparasite may be more adaptable to different conditions. While a salt‐sensitive ecotype of Arabidopsis was used in this experiment, it may be worthwhile to evaluate how host–parasite relations would change with a more salt‐tolerant ecotype (e.g., Landsberg erecta [Ler] or Wasilewskija [Ws]; Jha et al. 2010) or salt‐tolerant host species. There is some evidence to suggest that plant abiotic stress tolerance may be correlated with resistance against biotic stresses, although this was only reported in relation to biotic stresses other than parasitic plant infection (pathogen attack: Wiese et al. 2004; herbivory: Shafi et al. 2024). Future studies on other abiotic stress and a broader range of host species may help to determine if there are other cross‐resistances between abiotic responses and plant parasitic interactions.

Plant parasitism is traditionally viewed as dichotomous in its distinction of host fates in host–parasite relationships—plants that are naturally susceptible to parasitism (i.e., hosts) are generally assumed to perform poorly, providing a greater benefit to the parasite. Here, we demonstrated a bidirectional interaction between plant parasitism and salt stress, which was facilitated by changes occurring in a common physiological process: transpiration, which culminated in increased parasite salt sensitivity, as well as reduced parasitic benefits. Summarily, salinity stress can suppress parasitism in facultative root hemiparasites by repressing transpiration‐driven resource abstraction via salt‐induced stomatal closure within parasite leaves.

## Author Contributions

C.F.F.: Conceptualisation, methodology, validation, formal analysis, investigation, data curation, writing – original draft, writing – review and editing, visualisation, funding acquisition. M.A.: Investigation. E.Y. Investigation. L.J.I.: Conceptualisation, resources, writing – review and editing, supervision.

## Funding

This work was supported by the Japan Science and Technology Agency: Support for Pioneering Research Initiative by the Next Generation (JST‐SPRING) Fellowship, Grant Number: JPMJSP2124. Research was sponsored by the U.S. Army Research Office and accomplished under cooperative agreement W911NF‐19‐2‐0026 for the Institute for Collaborative Biotechnologies.

## Disclosure

No AI tools were used during the writing and revisions of this manuscript.

## Supporting information


**Figure S1:** Shoot nitrogen concentrations of host Arabidopsis and parasite *Phtheirospermum japonicum* (Experiment 1).
**Figure S2:** Leaf sodium [Na^+^] and potassium [K^+^] concentration of *Phtheirospermum* in Experiment 2, including host‐free attached (HFA) groups.
**Figure S3:** Parasite *Phtheirospermum japonicum* stomatal conductance (*g*_*s*_) measured 2.5, 3.5 and 5 h after foliar ABA application (Experiment 3).
**Figure S4:** *Phtheirospermum* stomatal conductance time‐series with host‐free attached (HFA) group (Experiment 2).
**Figure S5:** Phytohormone analysis of lyophilised *Phtheirospermum* leaf samples.
**Figure S6:** Experimental set‐up of haustoria counting experiment to test for the effect of salt on haustoria number.
**Figure S7:** Time‐series of haustoria counting experiment and haustoria micrographs.
**Table S1:** Results of three‐way ANOVA for parasite *Phtheirospermum* and Arabidopsis biomass (Experiment 1).
**Table S2:** Results of three‐way ANOVA for parasite *Phtheirospermum* leaf chlorophyll concentration [Chl] and linear electron flow (LEF) (Experiment 1).
**Table S3:** Results of three‐way ANOVA for parasite *Phtheirospermum* and host Arabidopsis leaf nitrogen concentration [N] (Experiment 1).
**Table S4:** Results of repeated measures ANOVA for parasite *Phtheirospermum* stomatal conductance (*g*_*s*_) (Experiment 2).
**Table S5:** *Phtheirospermum* shoot dry weight, relative water content and chlorophyll concentration and ANOVA analysis results (Experiment 2).
**Table S6:** Results of two‐way ANOVA for parasite *Phtheirospermum* phytohormone measurements.
**Methods S1**. Decapitation treatment (Experiment 2.1).
**Methods S2**. Phytohormone analysis (Experiment 2.2).
**Methods S3**. Haustoria Counting Time Series (Experiment S1).

## Data Availability

All primary data to support the findings of this study are openly available in Zenodo at https://doi.org/10.5281/zenodo.15758090.
