# Supplementary material for: Salt‐Induced Stomatal Closure Suppresses Parasitism by Phtheirospermum japonicum
Source: Physiol Plant. 2025 Nov 27;177(6):e70657. doi: 10.1111/ppl.70657 (PMC12660547; doi:10.1111/ppl.70657)
Supplement: Supplementary file 1 — Figure S1: Shoot nitrogen concentrations of host Arabidopsis and parasite Phtheirospermum japonicum (Experiment 1). Figure S2: Leaf sodium [Na+] and potassium [K+] concentration of Phtheirospermum in Experiment 2, including host‐free attached (HFA) groups. Figure S3: Parasite Phtheirospermum japonicum stomatal conductance (g s ) measured 2.5, 3.5 and 5 h after foliar ABA application (Experiment 3). Figure S4: Phtheirospermum stomatal conductance time‐series with host‐free attached (HFA) group (Experiment 2). Figure S5: Phytohormone analysis of lyophilised Phtheirospermum leaf samples. Figure S6: Experimental set‐up of haustoria counting experiment to test for the effect of salt on haustoria number. Figure S7: Time‐series of haustoria counting experiment and haustoria micrographs. Table S1: Results of three‐way ANOVA for parasite Phtheirospermum and Arabidopsis biomass (Experiment 1). Table S2: Results of three‐way ANOVA for parasite Phtheirospermum leaf chlorophyll concentration [Chl] and linear electron flow (LEF) (Experiment 1). Table S3: Results of three‐way ANOVA for parasite Phtheirospermum and host Arabidopsis leaf nitrogen concentration [N] (Experiment 1). Table S4: Results of repeated measures ANOVA for parasite Phtheirospermum stomatal conductance (g s ) (Experiment 2). Table S5: Phtheirospermum shoot dry weight, relative water content and chlorophyll concentration and ANOVA analysis results (Experiment 2). Table S6: Results of two‐way ANOVA for parasite Phtheirospermum phytohormone measurements. Methods S1. Decapitation treatment (Experiment 2.1). Methods S2. Phytohormone analysis (Experiment 2.2). Methods S3. Haustoria Counting Time Series (Experiment S1). [file PPL-177-e70657-s001.pdf]

Title: Salt-induced stomatal closure suppresses parasitism by *Phtheirospermum japonicum*

Author(s): Frederica, Clarissa F.; Asahina, Masashi; Yumoto, Emi and Irving, Louis J.

Supplementary Methods

- **Supp. Methods S1:** Decapitation treatment (Experiment 2.1)
- **Supp. Methods S2:** Phytohormone analysis (Experiment 2.2)
- **Supp. Methods S3:** Haustoria Counting Time Series (Experiment S1)

Supplementary Figures

- **Supp. Fig S1:** Shoot nitrogen concentrations of host Arabidopsis and parasite *Phtheirospermum japonicum* (Experiment 1).
- **Supp. Fig S2:** Leaf sodium [Na^+] and potassium [K^+] concentration of *Phtheirospermum* in Experiment 2, including host-free attached (HFA) groups.
- **Supp. Fig S3:** Parasite *Phtheirospermum japonicum* stomatal conductance (g_s) measured 2.5, 3.5 and 5 h after foliar ABA application (Experiment 3).
- **Supp. Fig S4:** *Phtheirospermum* stomatal conductance time-series with host-free attached (HFA) group (Experiment 2).
- **Supp. Fig S5:** Phytohormone analysis of lyophilized *Phtheirospermum* leaf samples.
- **Supp. Fig S6:** Experimental set-up of haustoria counting experiment to test for the effect of salt on haustoria number.
- **Supp. Fig S7:** Time-series of haustoria counting experiment and haustoria micrographs.

Supplementary Tables

- **Supp. Table S1:** Results of three-way ANOVA for parasite *Phtheirospermum* and Arabidopsis biomass (Experiment 1).
- **Supp. Table S2:** Results of three-way ANOVA for parasite *Phtheirospermum* leaf chlorophyll concentration [Chl] and linear electron flow (LEF) (Experiment 1).
- **Supp. Table S3:** Results of three-way ANOVA for parasite *Phtheirospermum* and host Arabidopsis leaf nitrogen concentration [N] (Experiment 1).
- **Supp. Table S4:** Results of repeated measures ANOVA for parasite *Phtheirospermum* stomatal conductance (g_s) (Experiment 2).
- **Supp. Table S5:** *Phtheirospermum* shoot dry weight, relative water content and chlorophyll concentration and ANOVA analysis results (Experiment 2).
- **Supp. Table S6:** Results of two-way ANOVA for parasite *Phtheirospermum* phytohormone measurements.

Supplementary Methods

Supplementary Methods S1: Decapitation treatment (Experiment 2.1)

A fifth treatment group containing 8 replicates was established alongside the four main groups described in the main text methods (Experiment 2), following the same growing protocol and treatment regime. In brief, host Arabidopsis and *Phtheirospermum* parasite were allowed to germinate and establish within the split-root set-ups as shown in Fig 2a, with root bridges between the host-only and interaction compartment to allow for host attachment. After five weeks of establishment, 5 ml of 100 mM NaCl was fed to the parasite-only compartment thrice a week for a further six weeks (42 d) of treatment. 10 d after the start of the salt treatment, day 10 stomatal conductance measurements were measured alongside the 4 treatment groups (Unattached, 'UA'/ Attached, 'A' x No-salt / Parasite-Salt, 'PS'). After stomatal conductance measurements were taken, all aboveground shoots of host Arabidopsis in this fifth treatment group were excised at the soil level to produce host-free attached (HFA) parasites.

HFA parasites were continually maintained with the rest of Experiment 2 for the remaining 32 d of salt treatment. During this period, no nutrient solution was given to the host-only sections. Stomatal conductance measurements of HFA parasites were taken alongside the other groups at day 20, 30, and 40 of salt treatment, and *Phtheirospermum* plants were harvested identically on day 42 of salt treatment (77 d since start of experiment). Parasite shoot sodium and potassium concentration of the HFA quantified following the same methods described in the main methods. HFA parasite stomatal conductance data is plotted alongside the other experiment 2 treatment groups in Supplementary Fig S4.

The objectives of this treatment group were as follows:

1) To test if high stomatal conductance rates of attached parasites can be maintained after host removal.
2) To test if increased salt uptake observed in attached salt-treated parasites is linked to elevated stomatal conductance rates associated with host attachment.

Statistical analyses

A one-way ANOVA of the effect of attachment (Unattached vs Attached vs Host-free attached) was conducted the shoot sodium and potassium concentrations of parasite-salt (PS) groups, followed by Tukey's post-hoc tests to determine differences between the three groups. Results of sodium and potassium salt concentrations are reported under Supplementary Fig S2.

Supplementary Methods S2: Phytohormone analysis (Experiment 2.2)

Decreased stomatal conductance was observed in salt-treated parasites in Experiment 2. To examine if parasite stomatal closure under salt conditions was accompanied by changes in leaf phytohormone concentrations, parasite shoot phytohormone concentrations were quantified. In particular, we wanted to check for changes in abscisic acid (ABA) due to its well-known role in regulating leaf stomatal behaviour, as well as its anomalous behaviour in root hemiparasites. We also measured additional phytohormones such as jasmonic acid (JA) and its derivatives, as well as cytokinins to obtain a comprehensive phytohormone profile of *Phtheirospermum* parasite under attached vs unattached, no-salt vs salt conditions.

Set-up

To prepare samples for the phytohormone analysis, six treatment groups were established in a 2 (attachment, unattached vs attached (UA/A)) x 3 (treatment, no-salt vs host-salt vs parasite-salt (No-salt/HS/PS) factorial design. Plants were grown identically as described in experiment 2 with a total establishment period of 35 d, followed by a further 42 d of treatment. At harvest, approximately 75 to 100 mg of *Phtheirospermum* leaves (per replicate) was collected, weighed, and immediately stored in -30 ºC freezer. At a later date, leaf samples were freeze-dried at -80 ºC for 24 h (EYELA FDU-2200, Tokyo Rikakikai Co. Ltd, Tokyo, Japan). Final sample dry weights were approximately 12 to 25 mg per sample.

Plant hormone analysis using LC-MS/MS

Phytohormone analysis was conducted at Teikyo University Advanced Instrument Analysis Center (Japan) using LC-MS/MS as previously described (Yamada et al., 2022)[1], with several modifications. Briefly, lyophilized leaf samples were suspended in 1 mL of 80% (v/v) aqueous methanol, with 2 ng of D_5-IAA, D_6-ABA, D_2-JA, $^{13}C_6$-JA-Ile, D_2-GA_1, D_2-GA_4 and 0.5 ng each of D_5-tZ, D_5-tZOG, D_5-tZROG, D_5-tZR, D_6-iP, and D_6-iPR added as an internal standard. Internal standards were purchased from Olchemim Ltd. (Olomouc, Czech Republic).

The samples were homogenized, and the supernatant was loaded onto a C18 column (Bond Elut C18, 100 mg/3 mL, Agilent Technologies, Palo Alto, CA, USA), which was equilibrated with 3 mL of 80% MeOH. The extract was collected, concentrated using a centrifugal evaporator, and resuspended in approximately 100 µL of 50 % (v/v) MeOH, and then 1 to 2 µL of the extract was injected into an Agilent 1200 high-performance liquid chromatography system coupled to an Agilent 6460 triple quadrupole mass spectrometer equipped with an ESI source (Agilent Technologies), ZORBAX Eclipse XDB-C18 reversed-phase column (50×2.1 mm, 1.8 µm particle size; Agilent Technologies). Plant hormones were monitored using multiple reaction monitoring (MRM). Data analysis was performed using MassHunter software (Agilent Technologies). The endogenous levels of plant hormones were calculated by the ratio of the analyte area to the area of the stable isotope-labeled internal standard of a known concentration.

Statistical Analyses

IAA, GA_1, GA_4, tZ, cZ, tZR, cZOG, cZROG and iP levels were not detectable in our samples and were thus excluded from any further analyses. Remaining phytohormone concentration data was analysed with two-way ANOVA with attachment (UA, A) and treatment (No-salt, host-salt 'HS', and parasite-salt 'PS') set as factors. Tukey's post-hoc tests were used to determine differences between groups. Analyses were done on R Ver. 4.5.0.

Supplementary Methods S3: Haustoria Counting Time Series (Experiment S1)

Set-up

Haustoria counting experiments were carried out in split-root set-ups as shown in Supplementary Figure S6. Boxes were filled with vermiculite, moistened with water, and then covered with a nylon mesh with a pore size of 59 µm that was stretched over the surface of the vermiculite. Arabidopsis seeds were directly sown on top of the mesh and moistened with dH_2O before being placed in a dark room at 4ºC for 48 h. After cold stratification, the boxes were returned to the growth chambers, and *Phtheirospermum* seeds were sown in the right compartment of the split root box as illustrated on Fig S4. The tops of the boxes were covered with cling wrap to prevent desiccation. 14 d after sowing, the cling wrap was removed, and 5 ml of Hoagland's solution was provided to both left and right compartments thrice a week. 28 d after sowing, boxes were tilted at an angle of 60º with cover plates facing downwards to promote root growth along the cover plate of the rhizotron set-up.

When each box contained at least five visible haustoria connections with the host (approximately 5 weeks from start of experiment), present haustoria were counted (day 0 of treatment), and the box was randomly assigned to two groups: no-salt, or salt treatment. Salt treatment consisted of 2 ml of 100 mM NaCl injected 4 cm from the top of the box on the right compartment. A total of 45 boxes were initially prepared, but due to poor establishment and high mortality rates, only 7 replicates per group remained at the end of the experiment. The number of visible haustoria found in the right compartment were counted on days 0, 5, 10, and 15 of salt treatment.

Parasite roots were harvested and cleared in 10% KOH for 48 h at room temperature. After clearing, root samples were washed in dH_2O and stored in 50% ethanol at 4 ºC until staining. Cleared roots were stained with 0.1% safranin-red solution for 5 mins at room temperature. Excess stain was then washed off, and roots were further destained in 50% ethanol for another 24 h before viewing under a bright-field microscope (Omax, South Korea). Micrographs were taken with a 14 MP USB camera mounted on the microscope, connected to the ToupView software. Safranin-red stains lignified structures red and enabled clear visualization of the xylem bridge structure in *Phtheirospermum* haustoria. For each box, recovered and stained haustoria were individually counted and classified into two categories: 1) Presumably functional haustorial (PFH, from Li & Guan, 2008[2]), or 2) non-mature haustoria. Percentage of PFH was calculated by taking number of PFH divided by total number of counted haustoria.

Statistical Analyses

Relative haustoria increase was obtained by dividing haustoria number on Day 5, 10, 15 by initial haustoria number on Day 0, before being analysed by repeated measures ANOVA with day as a within-subjects factor and treatment as a between-subjects factor. %PFH was analysed using t-tests. Statistical analyses were done in R Version 4.5.0.

<u>*Supplementary Figures*</u>

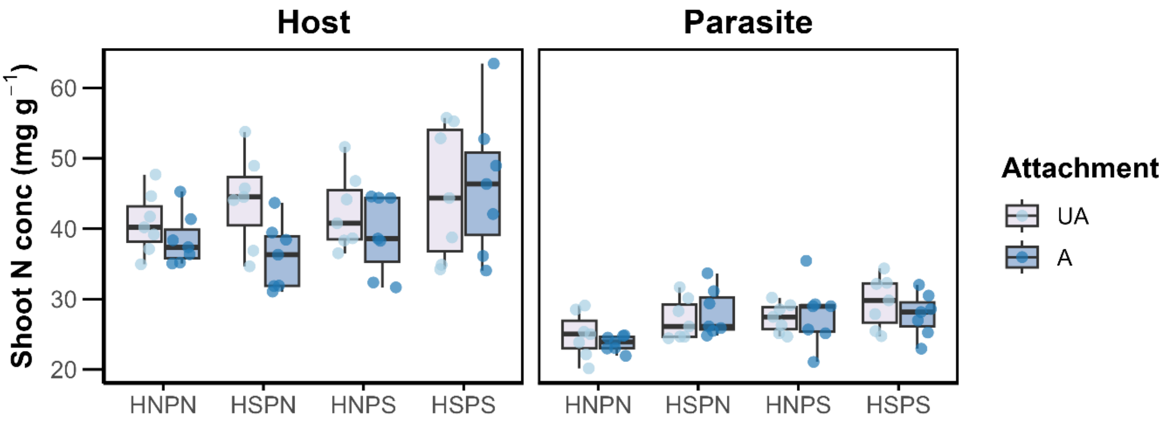

Supplementary Figure S1: Shoot nitrogen concentrations of host Arabidopsis and parasite *Phtheirospermum japonicum* (Experiment 1)

Each point represents an individual replicate. UA / A refer to unattached or attached plants. H/P= Host / Parasite, N/S = No-salt / Salt. n = 7.

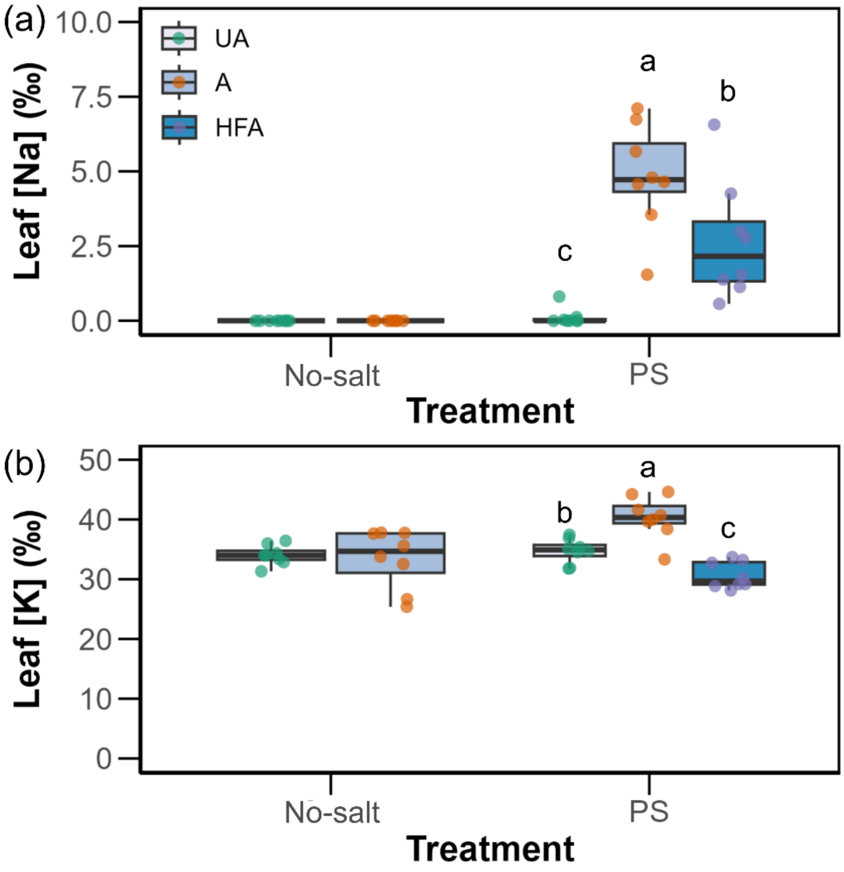

Supplementary Figure S2: (a) Leaf sodium [Na^+] and (b) potassium [K^+] concentration of *Phtheirospermum* in Experiment 2, including host-free attached (HFA) groups.

Each point represents an individual replicate. UA / A / HFA refer to unattached, attached, or host-free attached *Phtheirospermum* plants. PS refers to parasite-salt treatment. Sodium and potassium concentration was measured using inductively-coupled-plasma mass spectrometry (ICP-MS). Lowercase letters indicate significant differences between attachment groups in the PS treatment (Tukey's HSD). n = 8.

Trace levels of sodium were found in *Phtheirospermum* plants in the no-salt groups, while host attachment was observed to cause an increase in sodium levels in the PS treatments (see A, HFA PS groups). This corresponded with our findings in Experiment 1 (Fig 3a, main text). Furthermore, potassium levels were also elevated in attached PS treatments (Fig S2b), which would support the hypothesis that sodium accumulation was caused by increased transpiration rates, and by extension, increased overall root uptake and transfer of mineral ions into *Phtheirospermum* shoots.

Host decapitation on day 10 of the salt treatment resulted in moderate sodium levels in HFA-PS plants, corresponding with the restoration of parasite stomatal conductance to that of UA plants (see Supp. Fig. S5).

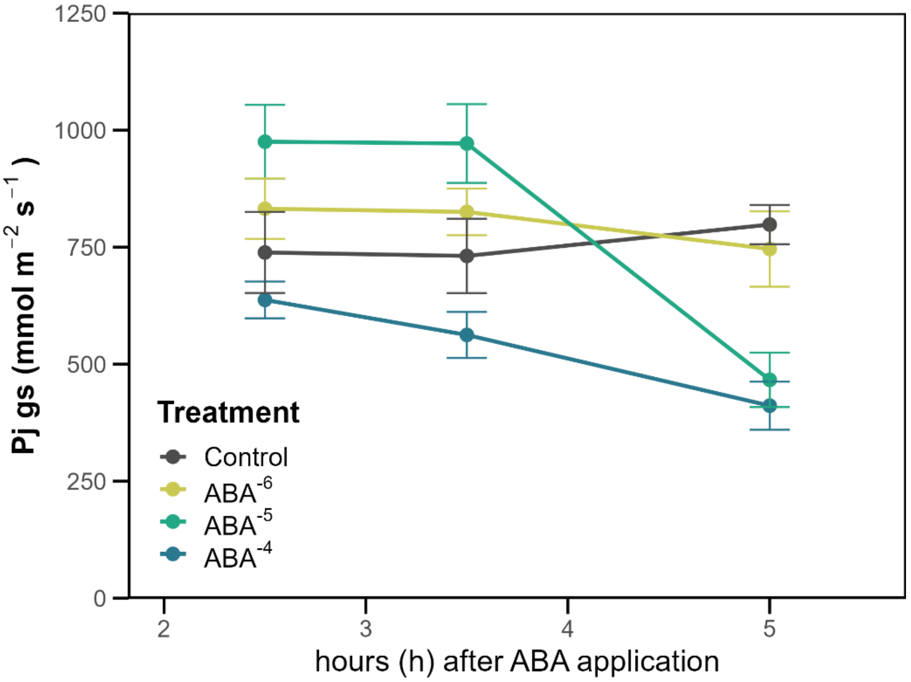

Supplementary Figure S3: Parasite *Phtheirospermum japonicum* stomatal conductance (g_s) measured 2.5, 3.5 and 5 h after foliar ABA application (Experiment 3)

In the control treatment, deionized water was applied on *Phtheirospermum* leaf surfaces rather than ABA dilutions. Points and error bars represent means and standard error at each time point, with different colours indicating different treatments. ^{15}N isotopic tracers were fed to hosts after g_s measurements were taken. n = 9 for control treatment, n = 10 for ABA treatments.

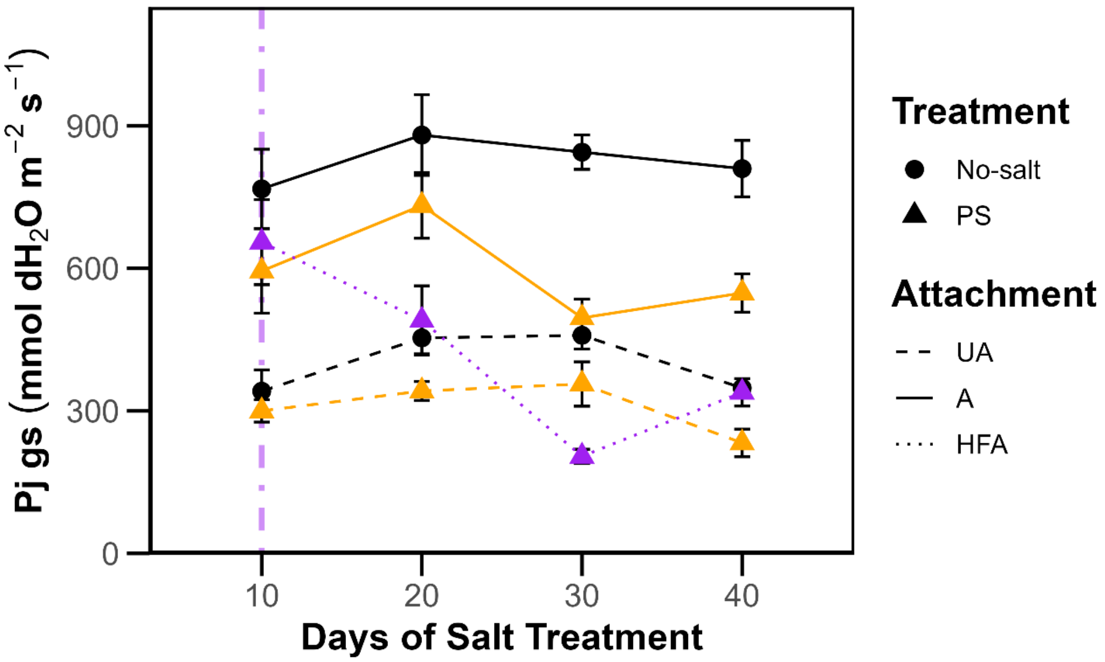

Supplementary Figure S4: *Phtheirospermum* stomatal conductance time-series with host-free attached (HFA) group (Experiment 2).

Points and error bars indicate mean and standard errors, dashed, solid and dotted lines represent unattached, attached and host-free attached groups respectively. Colours indicate treatments. The purple vertical line at day 10 indicates the day at which hosts were excised for the HFA groups.

Parasites in the HFA-PS group were allowed to form connections with the host, which we verified through day 10 g_s values, which showed that they were elevated relative to unattached (UA) parasites, but not significantly different from that of A-PS group. Hosts were then defoliated (as marked with the dot-dashed vertical line at day = 10) by excising the entire host rosette at the base. By day 20 of salt treatment, HFA-PS *Phtheirospermum* g_s had decreased to match that of UA parasites, which they maintained until the end of the experiment. n = 8.

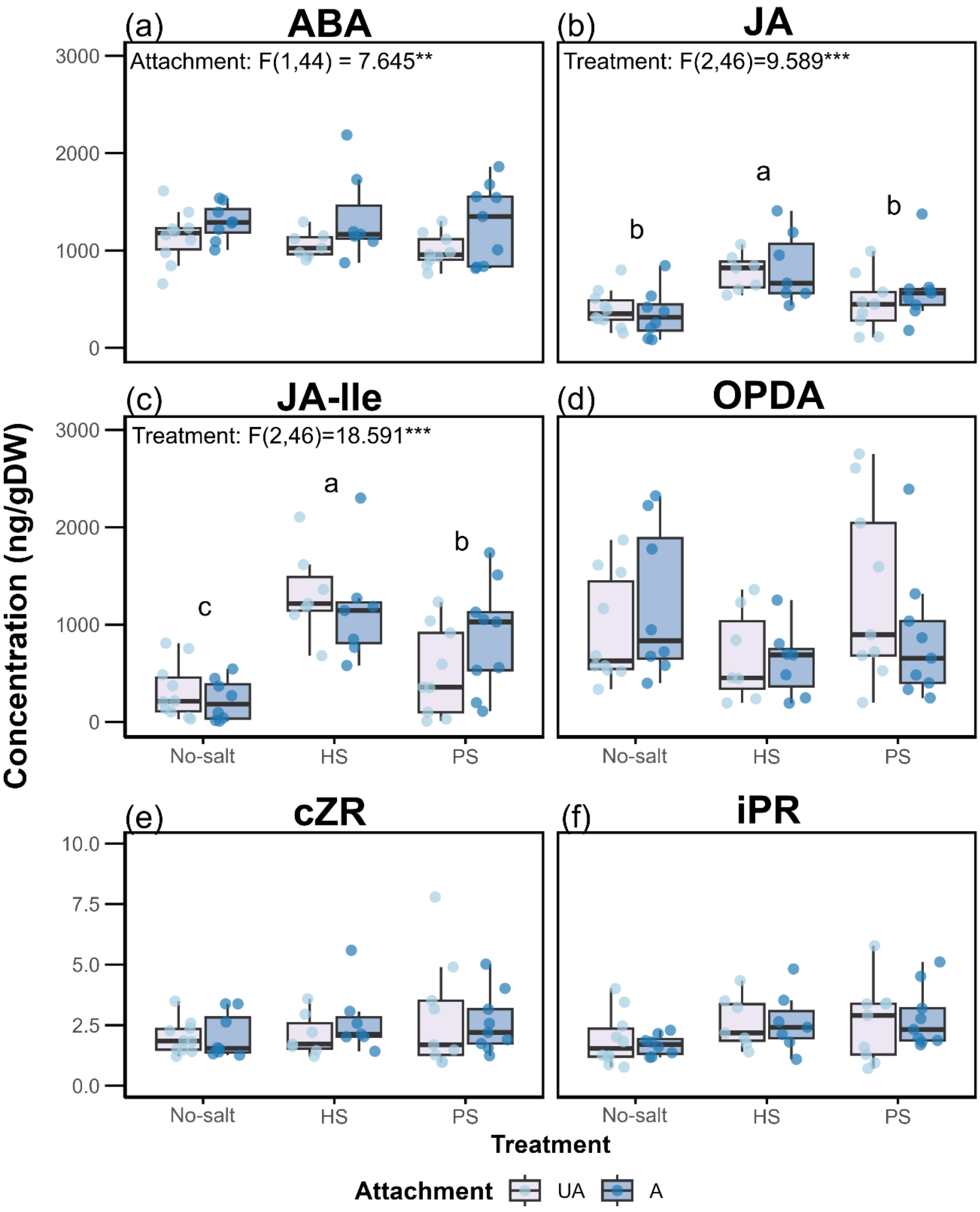

Supplementary Figure S5: Phytohormone analysis of lyophilized *Phtheirospermum* leaf samples.

Concentrations of abscisic acid (ABA), jasmonic acid (JA), JA-isoleucine (JA-Ile), JA-precursor oxylipin 12-oxophytodienoic acid (OPDA), and cytokinins cisZeatin-riboside (cZR) and isopentenyladenine-riboside (iPR). Analysis was carried out using liquid chromatography with tandem mass spectrometry (LC-MS) at Teikyo University (Japan). Points indicate individual replicates. n = 10, 7, 8 for no-salt, HS and PS group respectively. Only main effects were found for ABA, JA and JA-Ile, and no interactions were found (see Supplementary Table S6 for ANOVA results). ABA concentrations were significantly higher in attached *Phtheirospermum* parasites than unattached plants (a). Lowercase letters indicate significant differences ($p < 0.05$) between treatments (b, c).

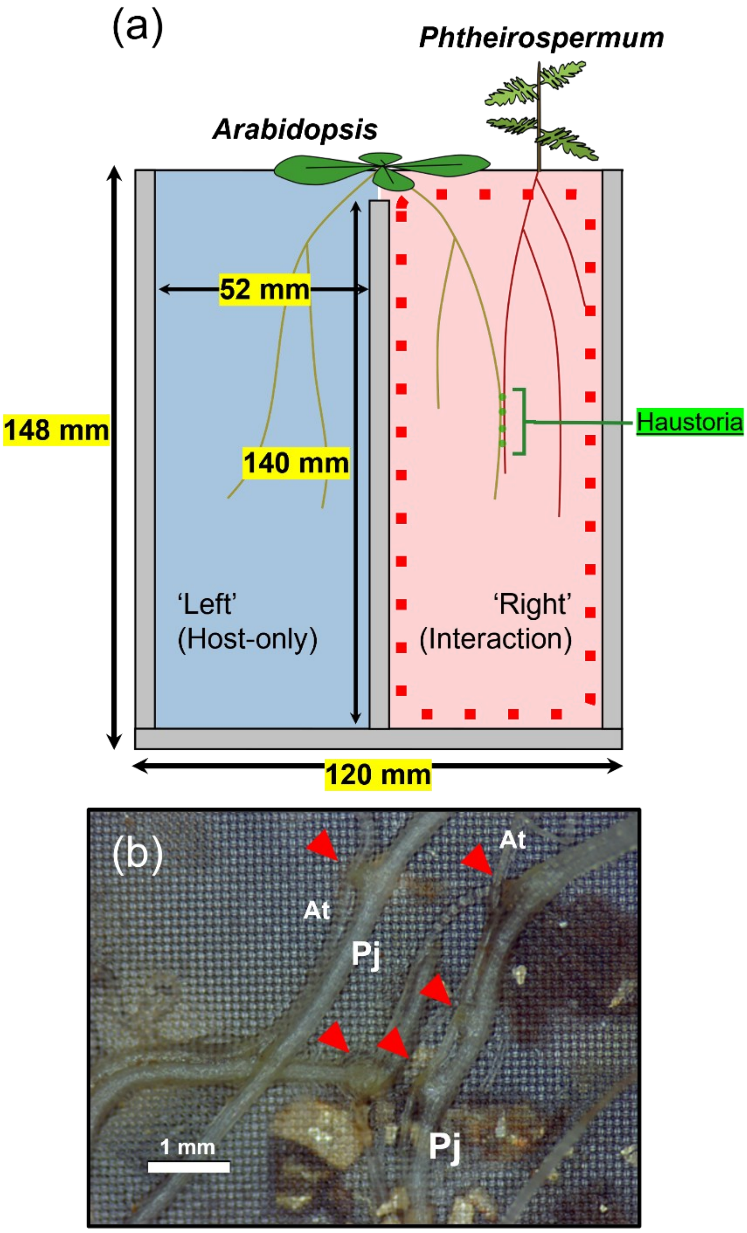

Supplementary Figure S6: Experimental set-up of haustoria counting experiment to test for the effect of salt on haustoria number.

(a) Split root-box set-up for *Phtheirospermum* parasite associated with Arabidopsis host. Boxes were constructed using 3 mm thick acrylic sheets (face / base plates) and 5 mm x 5 mm (cross section) acrylic bars. (b) *Phtheirospermum* haustoria connected to Arabidopsis host, growing over nylon mesh. Set-up diagram not drawn to scale. Red, dotted rectangle indicates where haustoria counts were taken. 'At / Pj' refer to Arabidopsis / *Phtheirospermum* roots respectively. Red triangles indicate haustoria connections.

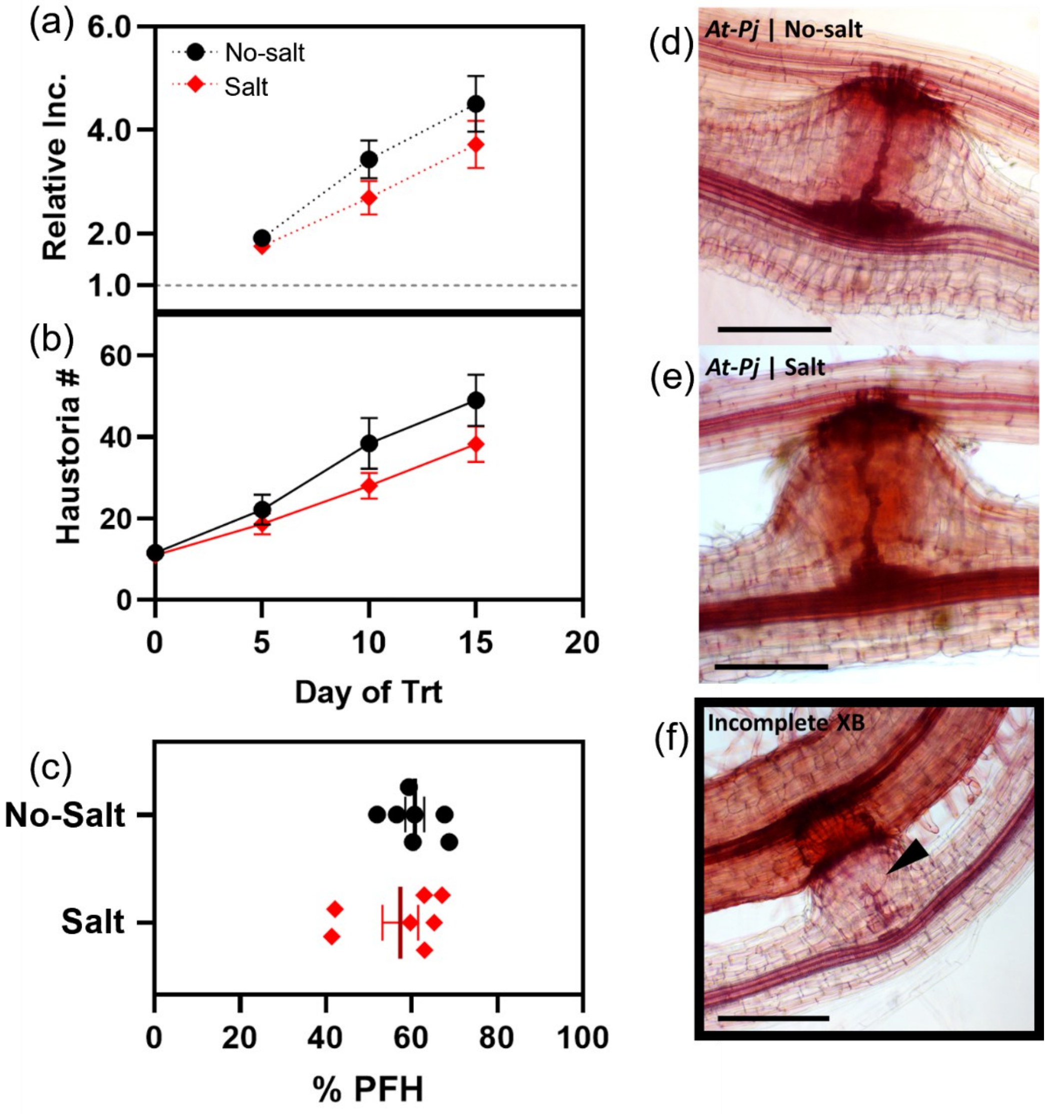

Supplementary Figure S7: Time-series of haustoria counting experiment and haustoria micrographs.

Time-series of (a) relative and (b) absolute increase in haustoria number over 15-d salt treatment period, and (c) percentage of haustoria with fully-functional xylem bridges. (d, e) shows micrographs of presumably functional haustoria (PFH) stained with Safranin-O, showing no differences in morphology between No-salt and Salt groups. (f) shows an example of a haustoria with an incomplete xylem bridge (marked by black triangle). Micrographs were taken at 100x magnification, scale bar = 200 µm.

Repeated measures ANOVA reported a significant main effect of day ($F_{(1,36)}$ = 6.779, P = 0.0133) on relative increase in haustoria number. Treatment had a marginally significant effect ($F_{(1, 36)}$ = 3.641, P = 0.0644). No interactions were reported.

Supplementary Tables

Supplementary Table S1: Results of three-way ANOVA for parasite *Phtheirospermum* and Arabidopsis biomass (Experiment 1).

F-values and P-values for the effects of attachment, parasite salt status (Ps) and host salt status (Hs) are reported here. $^+$ indicates parameter was log-transformed prior to analysis to pass Shapiro-Wilk's Test of Multivariate Normality. Significant effects are indicated in **bold**, while marginally significant effects (0.05 < P < 0.07) are indicated in *italics*.

		***Phtheirospermum* biomass**$^+$		***Arabidopsis* biomass**	
	df	**F**	**P**	**F**	**P**
Attachment	1	**61.688**	**<0.001**	*3.658*	*0.062*
Ps	1	0.469	0.497	2.435	0.125
Hs	1	0.006	0.940	0.569	0.454
Attachment x Ps	1	**8.669**	**0.005**	1.197	0.279
Attachment x Hs	1	0.006	0.940	1.302	0.260
Ps x Hs	1	1.530	0.222	0.025	0.874
Attachment x Ps x Hs	1	1.408	0.241	0.163	0.689
	48				

Supplementary Table S2: Results of three-way ANOVA for parasite *Phtheirospermum* leaf chlorophyll concentration [Chl] and linear electron flow (LEF) (Experiment 1).

F-values and P-values for the effects of attachment, parasite salt status (Ps) and host salt status (Hs) are reported here. Significant effects are indicated in **bold**.

		**[Chl]**		**Linear electron flow (LEF)**	
	df	**F**	**P**	**F**	**P**
Attachment	1	1.465	0.232	**15.951**	**<0.001**
Ps	1	**16.620**	**<0.001**	0.765	0.386
Hs	1	1.446	0.235	<0.001	0.994
Attachment x Ps	1	**8.417**	**0.006**	**7.833**	**0.007**
Attachment x Hs	1	**11.422**	**0.001**	**8.968**	**0.004**
Ps x Hs	1	**4.066**	**0.049**	1.456	0.233
Attachment x Ps x Hs	1	2.027	0.161	0.068	0.796
	48				

Supplementary Table S3: Results of three-way ANOVA for parasite *Phtheirospermum* and host Arabidopsis leaf nitrogen concentration [N] (Experiment 1).

F-values and P-values for the effects of attachment, parasite salt status (Ps) and host salt status (Hs) are reported here. Significant effects are indicated in **bold,** while marginally significant effects (0.05 < P < 0.07) are indicated in *italics*.

		***Phtheirospermum* [N]**		***Arabidopsis* [N]**	
	df	**F**	**P**	**F**	**P**
Attachment	1	0.212	0.647	3.097	0.085
Ps	1	**6.660**	**0.013**	*3.685*	*0.061*
Hs	1	**6.855**	**0.012**	2.323	0.134
Attachment x Ps	1	0.104	0.749	1.344	0.252
Attachment x Hs	1	0.001	0.973	0.034	0.855
Ps x Hs	1	1.773	0.189	1.564	0.217
Attachment x Ps x Hs	1	1.630	0.208	1.967	0.167
	48				

Supplementary Table S4: Results of repeated measures ANOVA for parasite *Phtheirospermum* stomatal conductance (g_s) (Experiment 2).

F-values and P-values for repeated measures ANOVA for the within-subjects effects of 'Day' (Day 10/20/30/40) and between-subjects effects 'Attachment' (UA/A) and treatment (No-salt/PS). ±indicates that Huynh-Feldt sphericity were conducted due to violations on the assumption of sphericity. Significant effects are indicated in **bold**.

		**Parasite g_s±**	
Within subjects	df	**F**	**P**
Day	2.667	**4.816**	**0.006**
Day x Attachment	2.667	1.859	0.150
Day x Treatment	2.667	1.296	0.282
Day x Attachment x Treatment	2.667	0.816	0.477
	74.664		
Between-Subjects			
Attachment	1	**125.811**	**<0.001**
Treatment	1	**26.481**	**<0.001**
Attachment x Treatment	1	**4.899**	**0.035**
	28		

Supplementary Table S5 *Phtheirospermum* shoot dry weight, relative water content and chlorophyll concentration and ANOVA analysis results (Experiment 2).

'UA/A' refers to unattached and attached groups respectively. 'PS' refers to parasite-salt treatment. (a) Numbers represent means (standard errors) of *Phtheirospermum* dry weight (DW), leaf relative water content (RWC) and chlorophyll concentrations [Chl] in respective treatments. (b) F-values and P-values for two-way ANOVA for effects 'Attachment' (UA / A) and treatment (No-salt / PS) is reported. Significant effects are indicated in **bold**. n = 8.

(a) Means (S.E)		**DW (mg)**		**RWC (%)**		**[Chl] (mg g^{-1} DW)**	
		**UA**	**A**	**UA**	**A**	**UA**	**A**
**No-salt**		560.1 (29.5)	1298.0 (91.0)	80.1 (0.3)	83.6 (0.2)	6.10 (0.39)	7.70 (0.45)
**PS**		604.9 (33.7)	1055.1 (80.9)	80.6 (0.8)	86.5 (0.7)	5.37 (0.52)	4.78 (0.42)
(b) ANOVA Results							
**Factors**	**df**	**F**	**P**	**F**	**P**	**F**	**P**
Attachment	1	**83.892**	**<0.001**	**112.859**	**<0.001**	1.287	0.266
Treatment	1	2.332	0.138	**14.708**	**<0.001**	**16.617**	**<0.001**
Attachment x Treatment	1	**4.920**	**0.035**	**7.566**	**0.010**	**6.031**	**0.021**
	28						

Supplementary Table S6 Results of two-way ANOVA for parasite *Phtheirospermum* phytohormone measurements.

F-values and P-values for effects of attachment (‘Att’: UA/A) and treatment (‘Trt’: no-salt/HS/PS) on phytohormone concentrations in *Phtheirospermum* leaves (Supplementary Figure S5) are reported here. Significant effects are indicated in **bold**. n = 10, 7, 8 for No-salt, HS and PS respectively.

(a)		**ABA**		**JA**		**JA-Ile**		**OPDA**	
	df	**F**	**P**	**F**	**P**	**F**	**P**	**F**	**P**
Att	1	**7.645**	**0.008**	0.523	0.473	0.460	0.501	0.285	0.596
Trt	2	0.392	0.678	**9.358**	**0.004**	**19.239**	**>0.001**	2.018	0.145
Att x Trt	2	0.262	0.770	0.446	0.643	1.802	0.177	1.372	0.264
	44								

(b)		**cZR**		**iPR**	
	df	**F**	**P**	**F**	**P**
Att	1	0.095	0.760	0.014	0.908
Trt	2	1.208	0.308	3.066	0.057
Att x Trt	2	0.387	0.681	0.152	0.859
	44				

[1] Yamada, K., Nakanowatari, M., Yumoto, E. et al. Spatiotemporal plant hormone analysis from cryosections using laser microdissection-liquid chromatography-mass spectrometry. *J Plant Res* 135, 377–386 (2022). https://doi.org/10.1007/s10265-021-01360-x

[2] Li, A.-R., & Guan, K.-Y. (2008). Arbuscular mycorrhizal fungi may serve as another nutrient strategy for some hemiparasitic species of Pedicularis (Orobanchaceae). *Mycorrhiza*, 18(8), 429–436. https://doi.org/10.1007/s00572-008-0196-z
